# Development of a New Benzofuran–Pyrazole–Pyridine-Based Molecule for the Management of Osteoarthritis

**DOI:** 10.3390/molecules28196814

**Published:** 2023-09-27

**Authors:** Somaia S. Abd El-Karim, Ahlam H. Mahmoud, Asmaa K. Al-Mokaddem, Noha E. Ibrahim, Hamad M. Alkahtani, Amer Alhaj Zen, Manal M. Anwar

**Affiliations:** 1 Department of Therapeutic Chemistry, Pharmaceutical and Drug Industries Research Institute, National Research Centre (NRC), El Bohouth St., Dokki, Cairo 12622, Egypt; ahlamhosnynrc@gmail.com; 2 Department of Pathology, Faculty of Veterinary Medicine, Cairo University, Cairo 12211, Egypt; asmaa.khairy@cu.edu.eg; 3 Department of Microbial Biotechnology, Biotechnology Research Institute, National Research Centre (NRC), El Bohouth St., Dokki, Cairo 12622, Egypt; nohaelsayed855@gmail.com; 4 Department of Pharmaceutical Chemistry, College of Pharmacy, King Saud University, Riyadh 11451, Saudi Arabia; ahamad@ksu.edu.sa; 5 Chemistry & Forensics Department, Clifton Campus, Nottingham Trent University, Nottingham NG11 8NS, UK; amer.alhajzen@ntu.ac.uk

**Keywords:** synthesis, osteoarthritis, antioxidant, pro-inflammatory mediators, histopathology

## Abstract

Osteoarthritis is a substantial burden for patients with the disease. The known medications for the disease target the mitigation of the disease’s symptoms. So, drug development for the management of osteoarthritis represents an important challenge in the medical field. This work is based on the development of a new benzofuran–pyrazole–pyridine-based compound **8** with potential anti-inflammatory and anti-osteoarthritis properties. Microanalytical and spectral data confirmed the chemical structure of compound **8**. The biological assays indicated that compound **8** produces multifunctional activity as an anti-osteoarthritic candidate via inhibition of pro-inflammatory mediators, including RANTES, CRP, COMP, CK, and LPO in OA rats. Histopathological and pharmacokinetic studies confirmed the safety profile of the latter molecule. Accordingly, compound **8** is considered a promising anti-osteoarthritis agent and deserves deeper investigation in future trials.

## 1. Introduction

Osteoarthritis (OA) is a degenerative condition that affects both surrounding tissues as well as bone cartilage. OA is a chronic arthropathy where the entire joint, including the cartilage, joint lining, ligaments, and the underneath bone, is impacted. Although OA can develop in any synovial joint in the body, it is more frequent in the spine, hands, and big joints (such as the knees and hips) [1,2,3]. The most common osteoarthritis symptom, joint discomfort, can cause locking or instability of the joint. Although this condition develops gradually, over time, it may result in joint failure [4]. The risk factors for this condition include heredity, feminine sex, aging, trauma from the past, inflammation, and obesity [5]. Over 100 million OA cases are expected to have been diagnosed globally by 2050 [6].

The pharmacological treatments that are currently available can only address the symptoms of OA and have unwanted side effects, particularly in older persons with frequent comorbidities [7,8,9]. Even though OA is not generally thought of as an inflammatory form of arthritis, there is evidence of subclinical low-grade inflammation across the entire joint and the inflammatory processes that are essential to the progression of the illness. The mediators released by bone and cartilage in addition to the synovium initiate the inflammatory processes [10,11].

Non-steroidal anti-inflammatory drugs (NSAIDs) are the best OA treatments for moderate to severe pain. They serve as COX-1 and COX-2 function suppressors, leading to a reduction in the formation of prostaglandin, resulting in anti-inflammatory, analgesic, and antipyretic effects [12,13]. NSAIDs are still the first-line treatment for illnesses involving chronic pain and inflammation. They are effective, but chronic use has been linked to gastrointestinal erosions, ulcerations, and bleeding, as well as cardiovascular complications. Therefore, it is both an enormous challenge and an urgent necessity to discover more effective anti-inflammatory medications with minimal toxicity with the goal of managing OA [14,15,16,17,18].

The benzofuran nucleus is a key component of many bioactive heterocycles. Many of the biological activities that benzofuran derivatives produce are substantial, including cytotoxic, antioxidant, antitubercular, anti-hyperglycemia, anti-Alzheimer’s, anti-inflammatory, and analgesic effects [18,19]. It has been reported that benzofuran-3(2*H*)-one derivative **I** exhibits a significant anti-inflammatory impact of managing different chronic inflammatory disorders. It effectively reduced tumor necrosis factor (TNF), interleukin 1 (IL-1), and IL-8 by 93.8%, 98%, and 71%, respectively. It also safely suppressed the NF-κB activity of human and murine macrophage cells [19].

**Figure 1:**
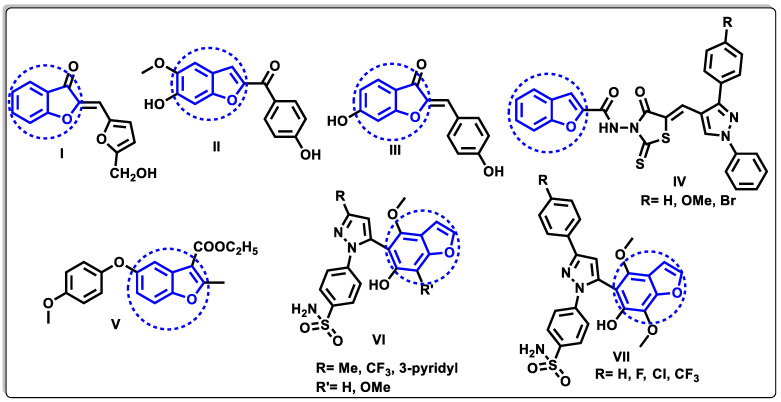
Various benzofuran-based derivatives of promising anti-inflammatory and analgesic activity.

Also, it has been reported that 2-aroylbenzofuran **II** and benzofuran-3-one derivative **III** produced promising anti-inflammatory impacts through excellent inhibition of NO production and NF-κB transcription activity, respectively, without cytotoxic effects [20,21]. Further studies showed that benzofuran compound **IV** produced a potent anti-inflammatory impact via COX-1, COX-2, and LOX suppression activity [22], whereas benzofuran compound **V** significantly suppressed the COX-2 effect [23]. Additionally, celecoxib analogs **VI** and **VII** bearing the benzofuran moiety demonstrated specific and robust COX-2 inhibition and were ineffective against COX-1 (IC_50_; >50 µM). Furthermore, both compounds’ gastric safety profiles were superior to those of the medication celecoxib [24] (Figure 1).

**Figure 2:**
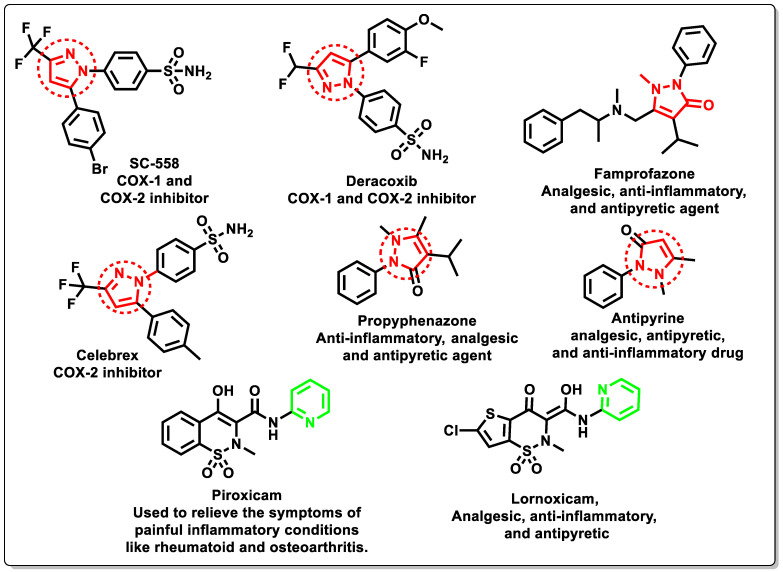
Different potent FDA-approved anti-inflammatory and analgesic drugs bearing pyrazole and pyridine pharmacophores.

The pyrazole nucleus, on the other hand, is a flexible lead molecule, and it has been observed that its derivatives have a wide range of biological functions. Antipyretic, anti-inflammatory, and analgesic activities are reported as major important activities of several pyrazole derivatives [25,26,27]. Figure 2 represents different potent pyrazole-based anti-inflammatory and analgesic drugs that were approved by the FDA [28,29].

Moreover, among the heterocycles that possess potent anti-inflammatory and analgesic activities is the pyridine ring. Piroxicam and lornoxicam are NSAIDs of the oxicam group. They are employed to overcome the symptoms of multiple painful inflammatory illnesses, including sciatica, surgery, osteoarthritis, and others. They function by halting the synthesis of endogenous prostaglandins, which mediate pain, stiffness, soreness, and edema [30,31,32]. 

A promising approach to drug development is the molecular hybridization strategy, which combines two or three pharmacophoric fragments into a single new scaffold. As compared to the individual parent molecules, the novel hybrid molecules appear to produce enhanced activity and selectivity profiles [33]. Accordingly, in a trial to improve the analgesic and anti-inflammatory effects of NSAIDs while eliminating their adverse effects with a superior gastrointestinal safety profile, this study deals with synthesizing the new benzofuran compound **8**.

**Figure 3:**
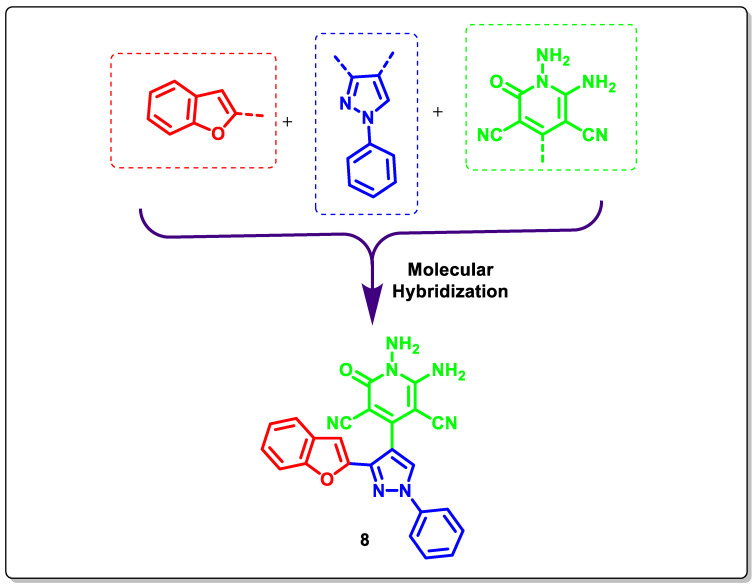
The design approach of the new benzofuran–pyrazole–pyridine-based compound **8**.

The latter compound is based on the conjugation of benzofuran, pyrazole, and substituted pyridine nuclei in the same scaffold, aiming to create a new NSAID with distinct anti-inflammatory and analgesic efficacy with few adverse effects and a high safety profile as an aid in osteoarthritis management (Figure 3). The generation of the target compound **8** was based firstly on the reaction of the precursor derivative, 2-acetylbenzofuran, with phenyl hydrazine to afford the corresponding 1-(benzofuran-2-yl)ethylidene-2-phenylhydrazone derivative (**3**), which was treated with phosphorus oxychloride and dimethyl formamide (Vilsmeier–Haack reaction) to form the corresponding carboxaldehyde derivative **4**. Moreover, the condensation reaction of **4** with malononitrile and 2-cyanoacetohydrazide in a basic medium resulted in the creation of the corresponding target compound, 1,6-diamino-4-(3-(benzofuran-2-yl)-1-phenyl-1*H*-pyrazol-4-yl)-2-oxo-1,2-dihydropyridine-3,5-dicarbonitrile (**8**) [34,35,36,37,38]. The substitution of the pyridine ring with NH_2_, CN, and C=O functionalities is expected to enhance the interaction of the compound in the active sites of various target proteins via different modes, such as hydrogen bonds.

This study deals with the evaluation of the antioxidant impact of the newly synthesized derivative **8** and its effect on the serum concentrations of pro-inflammatory mediators, including RANTES, CRP, COMP, CK, and LPO, in OA rats. In addition, compound **8** was subjected to histopathological and *in silico* studies to evaluate its pharmacokinetic properties and safety profile.

## 2. Results and Discussion

### 2.1. Chemistry

**Scheme 1:**
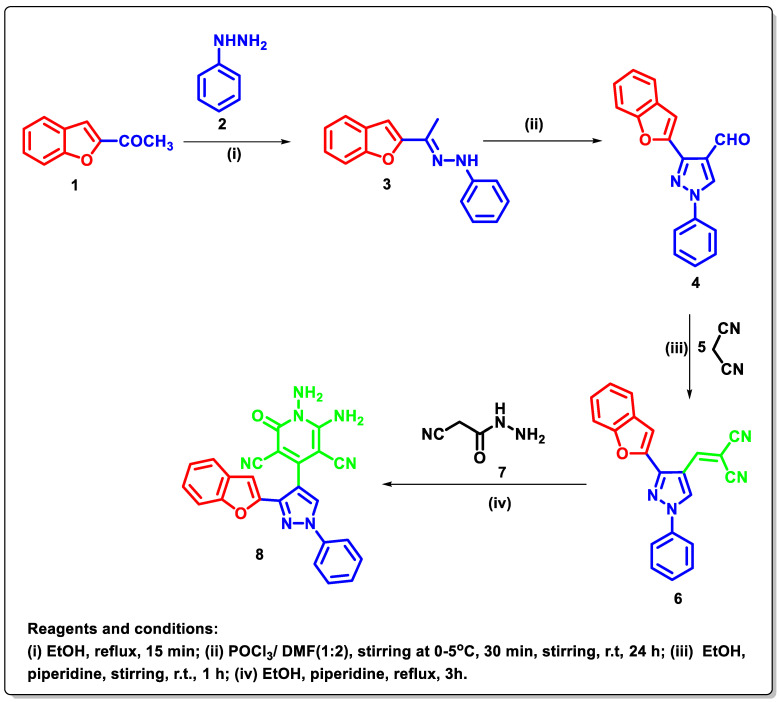
The synthetic approach of the benzofuran–pyrazole–pyridine-based derivative **8**.

The synthetic route utilized to prepare the new target benzofuran–pyrazole–pyridine derivative **8** is shown in Scheme 1. The key derivative, 2-acetylbenzofuran (**1**) [34], was allowed to react with phenyl hydrazine (**2**) in absolute ethanol to give 1-(benzofuran-2-yl)ethylidene-2-phenylhydrazone (**3**) [35]. The treatment of hydrazone derivative **3** with phosphorus oxychloride and dimethyl formamide (Vilsmeier–Haack reaction) yielded the corresponding carboxaldehyde derivative **4** [34,35,36,37,38], which was condensed with malononitrile (**5**) to form the corresponding methylene malononilrile derivative **6** [36]. The reaction of the latter malononilrile compound **6** with 2-cyanoacetohydrazide (**7**) in absolute ethanol containing a few drops of piperidine resulted in the creation of the corresponding target compound, 1,6-diamino-4-(3-(benzofuran-2-yl)-1-phenyl-1*H*-pyrazol-4-yl)-2-oxo-1,2-dihydropyridine-3,5-dicarbonitrile (**8**) (Scheme 1).

The IR spectrum of compound **8** displayed new strong absorption bands at 3393, 3315, and 3210 cm^−1^ related to the two amino groups, along with another band at 1661 cm^−1^ for the C=O group. The ^1^H NMR spectrum of **8** represented two singlet signals at δ 5.72 and 8.58 ppm due to NH_2_ protons. Meanwhile, the ^13^C NMR spectrum represented two signals at δ 76.00 and 88.18 ppm, referring to the two carbonitrile functionalities, and at δ 159.74 ppm, assignable to C=O of the pyridine nucleus. Additionally, the MS spectrum of the target compound **8** exhibited a molecular ion peak at *m*/*z* 433 in agreement with its molecular formula, C_24_H_15_N_7_O_2_.

### 2.2. Biological Evaluation

#### 2.2.1. DPPH Scavenging Activity

One of the significant factors in the pathophysiology of osteoarthritis is free radicals. The body will manufacture free radicals as osteoarthritis progresses, which will result in an imbalance between oxidative and antioxidative processes that is essential for chondrocyte injury. For older people who are more susceptible to developing OA and have a propensity for poor physical performance, antioxidants have been proposed as an alternative treatment. Recent studies have emphasized the necessity of antioxidants as a component of various treatment plans to counteract the damage that OA causes to the cartilage [39,40].

Accordingly, the new compound **8** was subjected to a DPPH scavenging assay to evaluate its antioxidant activity compared with ascorbic acid as a standard drug. Table 1 displays the proportion of the free radical scavenging activity at six distinct concentrations (10, 50, 100, 200, 500, and 1000 µg). The results showed that the activity of **8** increased in a dose-dependent manner. The most promising DPPH percentage of inhibition (84.4 ± 2.7%) was obtained using compound **8** at a concentration of 1000 µg compared with vitamin C’s DPPH percentage of inhibition of 97.14 ± 2.3% at the same concentration. Lower inhibition activities were obtained at lower concentrations.

#### 2.2.2. Identification of RANTES, CRP, COMP, LPO, CK, and Protein

Although the precise relationships between the biomarkers of inflammation and knee osteoarthritis (KOA) are still up for debate, inflammation is crucial to the development of OA. Pro-inflammatory proteins known as chemokines are crucial in inflammatory reactions and immune responses by chemoattracting and activating leukocytes [41]. In this study, the experimental rats were classified into five groups. The first group represents the healthy one or the negative control; the second group represents the rats that were injected with a single dose of mono-iodo acetate (MIA) to induce arthritis; the third group represents the rats that were injected with a single dose of MIA and supplemented orally with the tested compound **8** at a dose equivalent to 4.5 mg/kg/day; the fourth group was administered a single dose of MIA and **8** at a dose equivalent to 9 mg/kg/day; and the fifth group was administered a mixed dose of condrogen (4.5 mg/kg/day) and voltaren (9 mg/kg/day). The treatment was continued for 2 consecutive weeks. Then, the serum levels of RANTES, CRP, COMP, LPO, CK, and protein were determined in all tested animal groups.

RANTES (regulated on activation, normal T-cells expressed and secreted; also known as C-C chemokine ligand-5) induces the production of its own receptor, CCR-5, which supports an autocrine/paracrine pathway of the chemokine within the cartilage. High blood levels of RANTES have been associated with a faster progression of radiographic abnormalities, and high amounts of RANTES have been found in the synovial fluid (SF) obtained from RA patients. In comparison to peripheral blood, the percentage of lymphocytes and monocytes with CCR5 receptor expression is much greater in rheumatoid SF [42,43]. A serum analysis of OA rats for the determination of RANTES concentration demonstrated that a high RANTES concentration is associated with KOA incidence. According to Table 2, the RANTES concentration in the serum of KOA rats was 88.76 ± 1.8 Pg/mL, while those treated with compound **8** (4.5 mg/kg) exhibited an observed decrease in the RANTES concentration of 42.35 ± 1.43 Pg/mL, which is more potent than the result obtained using the reference drugs of a serum RANTES concentration of 66.02 ± 0.96 Pg/mL. Interestingly, the increase in the dose of compound **8** at a higher dose (9 mg) negatively affected the serum RANTES concentration, resulting in a higher concentration value of 55.86 ± 0.94 Pg/mL, but still more effective than both reference drugs.

Moreover, the pentameric protein called C-reactive protein (CRP) circulates in the plasma and is linked to inflammation, infection, and damage. Studies show a connection between CRP and problems such as diabetes, hypertension, and cardiovascular disease. Various studies exhibited a significant relationship between CRP concentration and KOA, whereas other studies investigated whether there was a significant correlation between CRP concentration and KOA incidence [42]. Additionally, a 535-kDa non-collagen protein called cartilage oligomeric matrix protein (COMP) has a high relation with the prevalence of KOA. The COMP serum levels in OA were different from those of healthy controls, according to certain evidence [44].

This study’s goal was to assess the relationship between the levels of CRP, the levels of serum COMP, and the occurrence/progression of KOA in light of these contradicting data. According to the results in Table 2, the serum levels of CRP and COMP in KOA rats were 2.5 ± 0.03 and 1.75 ± 0.07 ng/mL, respectively. When compound **8** (4.5 mg/kg) was administered to KOA rats, CRP and COMP serum levels fell to the same normal ranges as the negative control group: 2.23 0.05 ng/mL and 1.41 0.02 ng/mL. On the other hand, the treatment of KOA rats with the mixed dose of the standard drugs was less effective than compound **8**, producing COMP serum level concentrations of 3.25 ± 15.76 ng/mL, but more active in the case of CRP determination serum level concentrations of 1.85 ± 0.05 ng/mL. It could be noted that the increase in the treatment dose of compound **8** (9 mg/kg) retained the serum levels of both COMP and CRP (2.22 ± 0.11 ng/mL and 1.9 ± 0.01 ng/mL, respectively).

Furthermore, the compact enzyme creatine kinase (CK) is present in both the cystol and mitochondria of tissues, which have high energy requirements. Three CK isoenzymes, CK-MB (for cardiac muscle), CK-MM (for skeletal muscle), and CK-BB (for the brain), are tissue-specific. Excessive muscular exertion could initiate mechanical muscle damage of varying degrees [45]. The treatment of the KOA group with compound **8** (4.5 mg/kg) significantly suppressed the level of CK to reach 270 ± 4.16 ng/mL, which is very close to the normal concentration level in the control group, whereas the higher concentration of **8** exhibited a lesser suppression effect of the serum level: 308 ± 2.6 ng/mL. The CK level was greatly dropped using the dose of the standard drugs to reach 216 ± 0.03 ng/mL. Additionally, LPO is a byproduct of free radical lipid peroxidation, which affects the structure of cell membranes and can indirectly indicate the extent of free radical damage [46,47,48]. The treatment of KOA rats with compound **8** at both treatment doses (4.5 and 9 mg/kg) produced an approximately equal LPO serum level (0.76 ± 0.01 µM and 0.63 ± 0.017 µM, respectively) in comparison to the untreated groups with an LPO level of 0.87 ± 0.03 µM and a more potent impact than the reference drugs, which produced an LPO serum level of 1.06 ± 0.03 µM (Table 2).

Furthermore, the serum protein level slightly decreased using compound **8** (61 ± 2.27 g/mL) at the dose of 4.5 mg/kg, but unfortunately, the protein level significantly increased upon administration of the dose of 9 mg/kg. Compound **8** was less potent at both doses in comparison with the reference drugs, which reduced the protein level to 41.8 ± 2.02 g/mL.

It is obvious that the hybridization strategy of benzofuran, pyrazole, and substituted pyridine nuclei in the same architecture as in compound **8** led to significant anti-inflammatory activity via targeting two or more molecular proteins as an aid in osteoarthritis management. The presence of the heteroatoms nitrogen and oxygen might participate in interactions with the binding pockets of the proteins producing the evaluated biomarkers via hydrogen bonds, resulting in a reduction in their serum concentrations and a decrease in osteoarthritis severity.

In addition, the obtained data revealed that the increase in the concentration of compound **8** either decreased the target activity or had a negative impact. The increase in the concentration of the molecules might affect their orientation, thus negatively affecting their fitting in the active pockets of the proteins they act on.

#### 2.2.3. Histopathological Evaluation

Histopathological examination, a vital procedure, of the osteoarthritic joint tissues treated with compound **8** gives an important explanation about the effectiveness of the new compound to manage the disease as well as its safety profile in the examined tissues. Accordingly, the joints of the diseased rats were dissected and kept in neutral formalin at 10% for fixation. Decalcification of the joint tissue samples was performed in 20% EDTA, followed by routine processing protocols, and staining with hematoxylin and eosin (H&E) for light microscopy [47].

**Figure 4:**
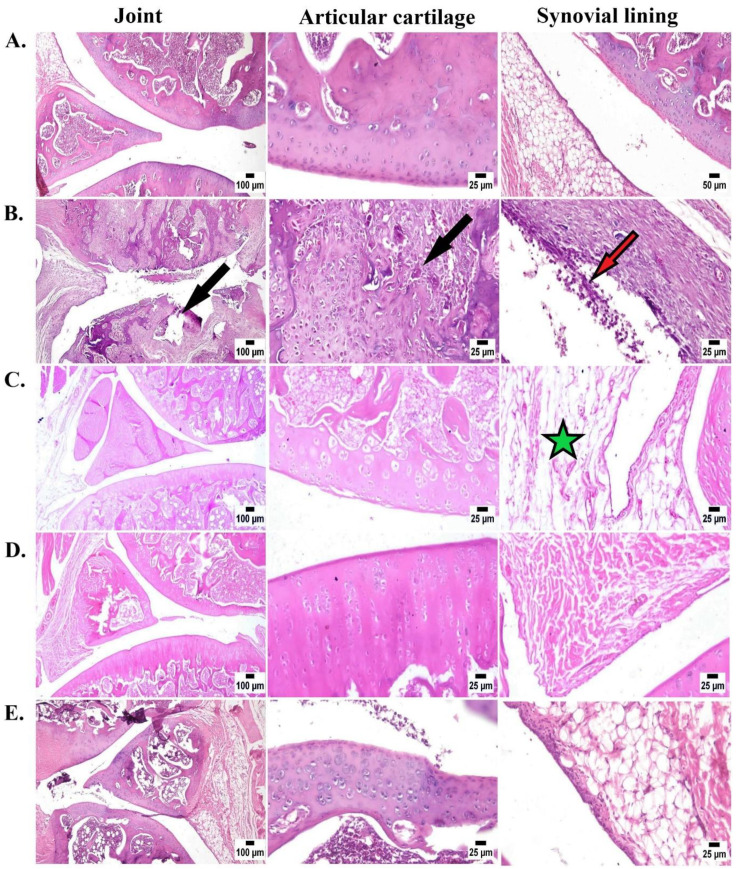
Photomicrograph of joint (H&E). (**A**) Control group showed normal joint structure. (**B**) Positive control showed necrotic articular cartilage (black arrows) and sloughing of synovial lining with mononuclear inflammatory cell infiltration (red arrow). (**C**) Low-dose compound **8** (4 mg/kg/day) showed apparently normal joints with mild periarticular edema. (**D**) Compound **8** (8 mg/kg/day) showed apparently normal joint. (**E**) Standard group showing apparently normal structure of joint.

Examining the control group exhibited normal structures of the joint capsule, synovial lining, and articular cartilage. Meanwhile, the diseased group showed marked histopathological alterations, including intense periarticular inflammatory cell infiltration, damage to articular cartilage, and sloughing of the synovial lining. On the other hand, the animal groups treated with compound **8** (4.5 or 9 mg/kg/day) exhibited a marked improvement, as all the examined joint sections were apparently normal with mild periarticular edema; no histopathological changes were detected in the examined sections. The treated groups showed marked improvement without any detectable alterations (Figure 4).

## 3. Computational Studies

### 3.1. Molecular Docking Study

**Figure 5:**
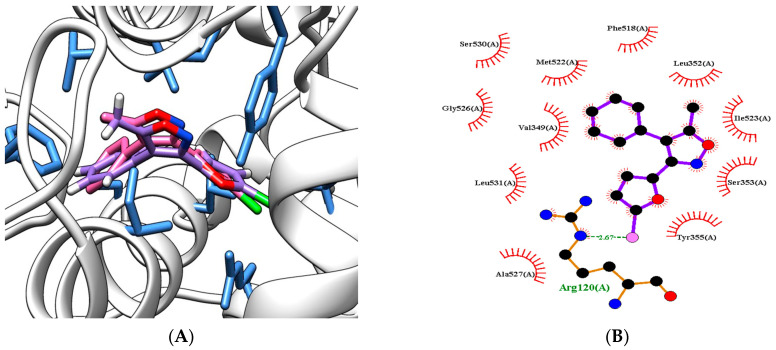
(**A**) Three-dimensional representation of the re-dock native ligand. The native ligand (purple, stick), and the re-docked ligand (purple, stick). (**B**) Ligplot+ diagram of the native ligand.

**Figure 6:**
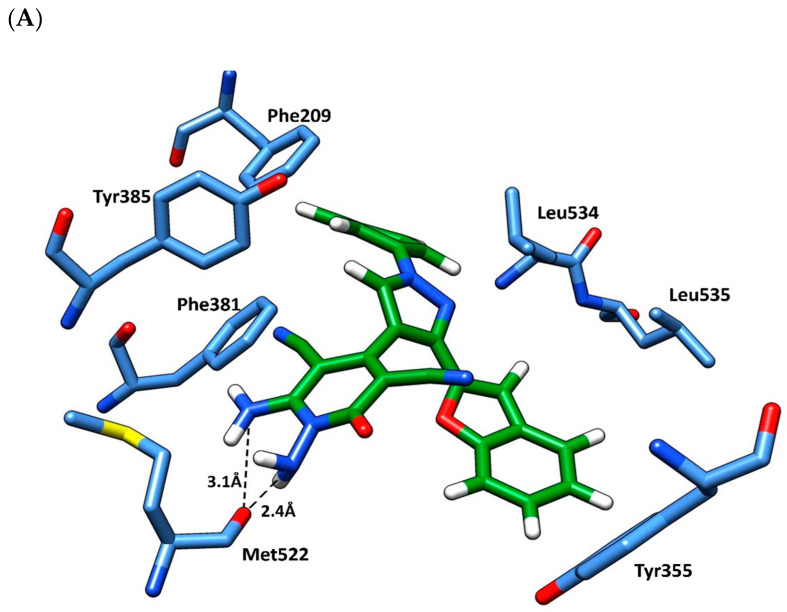

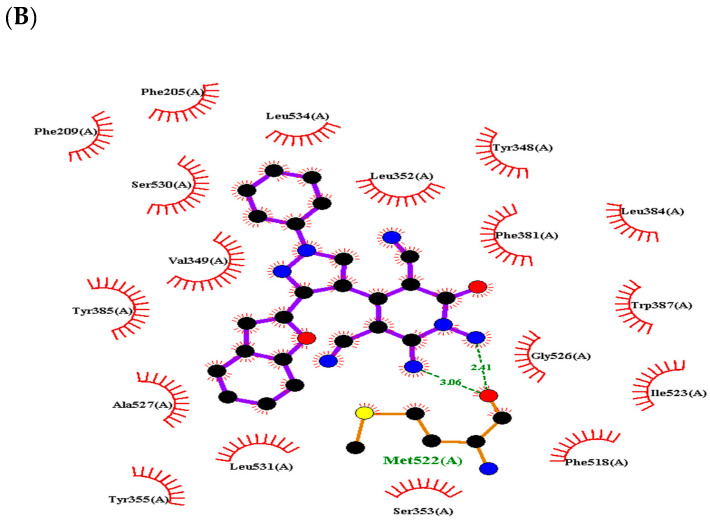
(**A**) Representation of re-dock compound **8** (within COX-1 binding site); compound **8** represented as the green stick structure. (**B**) Ligplot+ diagram of compound **8**.

Molecular modelling studies were carried out for compound **8** to study its mode of action. Cyclooxygenase-1 (COX-1) protein was selected for this study. Firstly, we re-docked the native ligand to validate the docking protocol. It was found that the re-docked pose produced a minimum RMSD < 1.0 Å against the original binding pose in the crystal structure (Figure 5A), and the re-docked pose occupied the same position as the original pose with a binding score of −6.8 Kcal/mol. These results indicated the reliability of the screening method that was used in this study. The interactions of the native ligand with COX-1 showed an H-bond between NH-Arg120 and with the F atom at a distance of 2.6 Å. The residues Met522, Ser353, and Tyr355 formed π–π interaction with the phenyl group (Figure 5B). Hydrophobic interactions were identified between Ile523, Ala527, Leu531, and the Isoxazole ring (Figure 5B, Table 3). Compound **8** showed a good binding mode within the COX-1 active site and a docking score of −9.5 Kcal/mol, which is better than the binding score of the native ligand (−6.8 Kcal/mol). The NH_2_ groups of compound **8** formed two H-bonds with OH-Met522 at distances (2.4 and 3.1 Å), respectively. The residues of Phe209, Phe381, Tyr385, and Tyr355 formed pi–pi interactions with the phenyl group and the pyrazole moiety of compound **8**. The benzofuran moiety formed hydrophobic interactions with Trp387, Leu352, Leu534, and Leu535 (Figure 6, Table 3). Further computational studies are still required.

### 3.2. ADMET Analysis

The relation between potency, pharmacokinetics, and toxicity is very crucial for the drug efficiency. Using pkCSM [48], Swiss ADME [49], and Data Warrior [48,50], the pharmacokinetics (absorption, distribution, metabolism, and excretion), drug-likeness, and toxicity features of the chemical compound **8** were assessed in this investigation. For a medicine to work effectively, potency, pharmacokinetics, and toxicity must interact.

Table 4 represents the drug-likeness properties and synthetic accessibility of compound **8**. It is evident that compound **8** satisfies Lipinski’s rule of five, which forecasts the drug’s oral bioavailability. The molecular weight of compound **8** is 500, hydrogen bond donors <5, hydrogen bond acceptor <10, and Clog *p* < 5. These parameters confirm the good oral bioavailability of compound **8**. Also, compound **8** passed the PAINS filtrate. The synthetic accessibility of compound **8** is close to 1, which indicates that it is easy to prepare.

Additionally, the biological effects and bioavailability of the drug depend greatly on absorption. The absorption of compound **8** in the human intestinal tract was high (89%) while Caco2 cells showed low permeability for compound **8** (Table 5). In addition, compound **8** is unsuitable for transdermal applications, according to the negative value of *in vitro* skin permeability (Table 5). In line with its anti-osteoarthritis efficacy and lack of requirement to cross the blood–brain barrier, compound **8** displayed low blood–brain barrier permeability and CNS permeability (Table 5). Moreover, compound **8** is not predicted to inhibit liver enzymes like CYPC1A2 and CYP2D6, but it is predicted to be a CYP3A4 substrate. The total clearance of compound **8** is 1.089 mL/min/kg, and it is not a substrate for the renal OCT transporter (Table 5). Furthermore, the toxicity assessments of compound **8** proved it is not mutagenic, tumorigenic, or a skin irritant, which indicates that compound **8** has a safe profile (Table 6).

## 4. Conclusions

In summary, based on the promising anti-inflammatory activity of benzofuran, pyrazole, and pyridine heterocycles, this study deals with the design and synthesis of the hybridized compound **8** of the latter ring systems in an effort to generate a new NSAID with significant anti-inflammatory and analgesic efficacy with minimum adverse effects and a high safety profile as an aid in osteoarthritis management, which was assessed on a model of knee osteoarthritis in rats.

The new compound demonstrated promising anti-osteoarthritis activity. The study exhibited that the mechanism of action of compound **8** is multifunctional via promising suppression of different inflammatory factors such as RANTES, CRP, COMP, and CK and controlling free radicals such as LPO. Histopathological examination of the diseased rats treated with compound **8** exhibited a marked improvement, as all the examined joint sections were apparently normal with mild particular edema and no histopathological changes. An ADMET analysis confirmed that compound **8** has promising drug-like properties and is easy to synthesize. Additionally, it adheres to Lipinski’s rule of five, a sign of strong oral bioavailability. Moreover, compound **8** showed a high absorption rate of human intestinal cells and low skin permeability. Interestingly, compound **8** showed a safe profile alongside negative mutagenic and tumorigenic effects and non-irritant effects on the skin.

The obtained results confirmed the promising target of the design strategy, and the therapeutic potential of the new compound **8** for managing osteoarthritis necessitates deeper studies and investigation in future trials.

## 5. Experimental Protocols

### 5.1. Chemistry

The instrumental devices used for measuring the melting points, spectral data (IR, mass, ^1^H NMR, and ^13^C NMR), and elemental analyses are detailed in Supporting Information.

2-Acetylbenzofuran (**1**), 1-(benzofuran-2-yl)ethylidene-2-phenylhydrazone (**3**), 3-(benzofuran-2-yl)-1-phenyl-1*H*-pyrazol-4-carboxaldehyde (**4**), and 2-((3-(benzofuran-2-yl)-1-phenyl-1*H*-pyrazol-4-yl)methylene)malononitrile (**6**) were prepared according to methods reported in the literature [34,35,36,37,38].

#### 5.1.1. Synthesis of 1-(1-(Benzofuran-2-yl)ethylidene)-2-phenylhydrazine (**3**)

The compound 1-(1-benzofuran-2-yl)ethanone (**1**) (1.60 gm, 0.01 mol) was dissolved in ethanol (25 mL) containing a catalytic amount of acetic acid in a round-bottom flask. Then, phenyl hydrazine (1.10 gm, 0.01 mol) was added dropwise. The mixture was stirred at room temp. for about 1 hr. After the completion of the reaction as indicated with TLC, the Schiff’s base **3** formed was isolated and recrystallized from ethanol in an 82% yield and m.p. 86–88 °C [35].

#### 5.1.2. Synthesis of 3-(Benzofuran-2-yl)-1-phenyl-1H-pyrazole-4-carboxyaldehyde (**4**)

In a round-bottom flask, phosphorus oxychloride (20 mL, 0.2 mol) was added dropwise with stirring to dimethyl formamide (150 mL) at 0–5 °C. Then, (1-benzofuran-2-yl)ethylidene-2-phenyl hydrazine (23.5 gm, 0.094 mol) was added portion-wise with continuous stirring. The reaction mixture was left overnight at room temperature, poured onto ice-cold water, and neutralized with an ammonium hydroxide solution (5%). The formed precipitate was filtered, dried, and recrystallized from acetic acid to give the title compound **4** in a 90% yield and m.p. 160–162 °C [36,37,38].

#### 5.1.3. Synthesis of 2-((3-(Benzofuran-2-yl)-1-phenyl-1H-pyrazol-4-yl)methylene)malononilrile (**6**)

A mixture of compound **4** (0.58 g, 0.002 mol) and malononitrile (**5**) (0.13 mL, 0.002 mol) in absolute ethanol (15 mL) containing a few drops of piperidine was warmed for 15 min and stirred for 1 hr at room temperature. The formed precipitate was filtered, dried, and recrystallized from absolute ethanol (99%) to give the title compound **6** in a 74% yield and m.p. 180–181 °C [36].

#### 5.1.4. Synthesis of 1,6-Diamino-4-(3-(benzofuran-2-yl)-1-phenyl-1H-pyrazol-4-yl)-2-oxo-1,2-dihydropyridine-3,5-dicarbonitrile (**8**)

A mixture of compound **6** (0.87 gm, 0.002 mol) and 2-cyanoacetohydrazide (**7**) (0.2 gm, 0.002 mol) in absolute ethanol (15 mL) containing a few drops of piperidine was refluxed for 3 h. The formed precipitate was filtered, dried, and recrystallized from absolute ethanol.

Yield = 85%; mp 269–270 °C; IR (*ν*_max_/cm^−1^): 3393, 3315, 3210 (2NH_2_), 2217 (CN), 1661 (CO); ^1^H NMR (300 MHz; DMSO-*d*_6_) *δ*_H_ 5.72 (s, 2H, NH_2_, D_2_O exchangeable), 7.17 (s, 1H, Ar-H), 7.27–7.47 (m, 3H, Ar-H), 7.59–7.70 (m, 4H, Ar-H), 8.01 (d, 2H, Ar-H, *J* = 7.8 Hz), 8.58 (br, 2H, NH_2_, D_2_O exchangeable), 9.11 (s, 1H, pyrazole proton) ppm; ^13^C NMR (75 MHz; DMSO-*d*_6_) *δ*_C_ 76.0, 88.1,105.3, 111.8, 115.8, 115.8, 116.7, 119.1, 122.0, 123.9, 125.6, 128.04, 128.6, 130.4, 139.1, 141.5, 148.5, 151.3, 154.6, 157.1, 159.7. MS, *m*/*z* (%): 433 [M^+^] (37), 434 [M^+1^] (19), 77 [due to C_6_H_5_] (100). Anal. calcd. for C_24_H_15_N_7_O_2_ (433.43): C, 66.51; H, 3.49; N, 22.62; found: C, 66.32; H, 3.22; N, 22.43.

### 5.2. Biochemical Study

#### 5.2.1. In Vivo Study

##### Animals

The experimental rats: thirty Wister albino rats of both sexes were used in this investigation. These animals were apparently healthy and weighed 150–200 g. Rats were housed in hygienic fiber-glass cages. They were obtained from the animal house of the National Research Center, Egypt. Animals were fed with commercial pellets. The rats were allowed free access to tap water and feed throughout the experiment. All animals were observed daily for signs of toxicity.

##### Knee Inflammation Induction in Rats

A single intra-articular injection (i.a.) of monosodium iodoacetate (MIA) was injected through the infra-patellar ligament into the joint space of the right knee of lightly anesthetized rats (3% isoflurane in O_2_ at 1.5 L/min). The single-dose i.a. injection of 3 mg of MIA (Sigma-Aldrich, St. Louis, MO, USA) was prepared by dissolving MIA (3 mg) in 50 µL of 0.9% physiological saline in a total volume of 50 μL of saline according to the methodology of Udo et al., 2016 [51]. All rats were kept under observation throughout the experiment.

##### Experimental Design

The animals were constructed into five groups of rats; each group contained six rats:

The first group represents the healthy group or the negative control.

The second group represents the rats who were injected with mono-iodo acetate (MIA) with a single dose.

The third group represents the rats who were injected with a single dose of mono-iodo acetate (MIA) and supplemented orally with the tested compound 8 of a dose equivalent to 4.5 mg/kg/day for 2 weeks.

The fourth group: Rats were injected with mono-iodo acetate (MIA) with a single dose and supplemented orally with compound 8 with a dose equivalent to 9 mg/kg/day for 2 weeks.

The fifth group: Rats were injected with 50 mg of MIA in a total volume of 50 μL of saline in the right knee, and then treated with standard drugs, Chondrogen (4.5 mg/Kg/day) mixed with voltaren (9 mg/Kg), orally for 2 weeks.

At the end of the treatment, blood samples were drawn from the animals by puncturing the retro-orbital venous plexus with a thin sterilized capillary tube under light anesthesia, using isoflurane as an inhaled anesthetic in a drop jar at a concentration of 4–5% then 2% for maintenance. Blood specimens were centrifuged to separate serum and stored at −80 °C until needed. Rats were then sacrificed using decapitation and joints were dissected.

#### 5.2.2. Measuring of Biological Parameters

##### DPPH Radical Scavenging Assay

Free radical scavenging ability of compound **8** was tested with a DPPH radical scavenging assay as described by Desmarchelier et al., 1997 [52]. More details are provided in Supporting Information.

##### Evaluation of RANTES, CRP, COMP, LPO, CK, and Protein in KOA Rats with ELISA

Rat regulated on activation in normal T-cells expressed and secreted (RANTES), rat creatine kinase (CK), rat lipid peroxide (LPO), rat C-reactive protein (CRP), and rat cartilage oligomeric matrix protein (COMP) were measured in rat serum with ELISA according to the instruction kits with catalogue nos. SL0616Ra, SL1186Ra, SL1340Ra, SL0202Ra, and SL1042Ra, respectively. Total protein was measured in rat serum with a calorimetric method (Biuret reagent), according to the instruction kit, from the spectrum and read at 560 nm.

#### 5.2.3. Histopathological Study

Joints were dissected and kept in neutral formalin (10%) for fixation. Decalcification of joint tissue samples was performed in 20% EDTA followed by a routine processing protocol and staining with hematoxylin and eosin (H&E) for light microscopy.

#### 5.2.4. Molecular Docking

##### Preparation of the Protein

The crystal structure of cyclooxygenase−1 and COX−1 (PDB:5U6X) complexed with 3-(5-chlorofuran-2-yl)-5-methyl-4-phenylisoxazole was downloaded from a protein data bank (https://www.rcsb.org/structure/5U6X, accessed on 5 September 2023). The protein was prepared according to the reported method [53]. Hydrogen atoms were added to the crystal structure. The water molecules and ions were removed, and the protonation state of each residue was determined at pH 7 using the PDB2PQR server (http://nbcr-222.ucsd.edu/pdb2pqr2.0.0/, accessed on 5 September 2023) [54]. The missing loop regions and missing residues were inserted with the MODELLER program. The protein was kept rigid during the docking.

##### Preparation of the Ligands

The native ligand and compound **8** were protonated, and partial charges were assigned with MOE software using the Gastiger method and it saved ligands as mol2 files [55]. The energies of ligands were minimized with the MMFF94 force field until an RMSD gradient of 0.05 kcal/mol Å. Both ligands were kept flexible during the molecular docking.

##### Molecular Modelling

To investigate the accuracy of the docking protocol, the native ligand was re-docked using the docking scheme. The docking studies were carried out using MOE software against the cyclooxygenase-1 complex (PDB:5U6X); the position of the native ligand was identified and used for docking studies of compound **8**. The Placement tool was implemented as Triangle Matcher. The scoring criteria were London dG, and the rescoring affinity dG was applied for refinement [56].

##### Compliance with Ethical Standards

All animal treatments were carried out in accordance with the National Research Centre’s (NRC) Guidelines for the Care and Use of Laboratory Animals in Dokki, Giza, Egypt (NRC), and were authorized by the MREC of the NRC (NRC no. 34412012023).

## Figures and Tables

**Table 1:** DPPH scavenging activity of the target compound **8**.

Concentrations	DPPH Scavenging Activity (%) of Ascorbic Acid	DPPH Scavenging Activity (%) of Compound 8
10	54.88 ± 3.1	17.93 ± 2.1
50	97.4 ± 2.1	14.02 ± 1.8
100	97 ± 2.3	11.59 ± 1.8
200	97 ± 1.7	23 ± 2.5
500	97.2 ± 1.9	50 ± 2.3
1000	97 ± 2.3	84.4 ± 2.7

**Table 2:** Serum levels of pro-inflammatory biomarkers RANTES, CRP, COMP, LPO, CK, and protein in KOA rats.

Groups	RANTES (Pg/mL)	CRP (ng/mL)	COMP (ng/mL)	CK (ng/mL)	LPO (µM)	Protein (g/mL)
1-Negative control	60.17 ± 0.85 (2 ***, 3 ***, 4 *, 5 **)	2.10 ± 0.01 (2 ***)	1.46 ± 0.012 (2, 4, 5) ***	262 ± 5.2 (2 ***, 4 **, 5 ***)	0.76 ± 0.009 (2, 4, 5) ***	53.4 ± 0.64 (4 ***)
2-Positive control	88.76 ± 1.8 (1, 3, 4, 5) ***	2.51 ± 0.03 (1 ***, 3 **, 4 **, 5 **)	1.75 ± 0.07 (1 ***, 3 ***, 4 *)	519 ± 6 (1, 3, 4, 5) ***	0.87 ± 0.03 (1, 3, 4, 5) ***	63.43 ± 1.82 (4 ***, 5 **)
3-Low dose	42.35 ± 1.43 (2, 4, 5) ***	2.23 ± 0.05 (2 **)	1.41 ± 0.02 (2, 4, 5) ***	270 ± 4.16 (2 ***, 3 **, 5 ***)	0.76 ± 0.01 (2, 4, 5) ***	61 ± 2.27 (4 ***, 5 *)
4-High dose	55.86 ± 0.94 (1 *, 2 ***, 3 ***, 5 ***)	2.22 ± 0.11 (2 **)	1.90 ± 0.01 (1 ***, 2 *, 3 ***)	308 ± 2.6 (1 **, 2 ***, 3 **)	0.63 ± 0.017 (1, 2, 3, 5) ***	120.45 ± 9.16 (1, 2, 3, 5) ***
5-Ref. drug	66.02 ± 0.96 (1 **, 2 ***, 3 ***, 4 ***)	1.85 ± 0.05 (1, 3) ***	3.25 ± 15.76 (1, 2, 3) ***	216 ± 0.03 (2 **)	1.06 ± 0.03 (1, 2, 3, 4) ***	41.8 ± 2.02 (2 **, 3 *, 4 ***)

Results: mean ± SE, * *p* < 0.05, ** *p* < 0.01, *** *p* < 0.001. Reference drugs: mixed doses of condrogen (4.5 mg/kg/day) and voltaren (9 mg/kg/day).

**Table 3:** Docking score and interactions of native ligand and compound **8**.

	Docking Scores (Kcal/mol)	Residues of Proteins	Moieties of Compounds	Type of Interactions
Native ligand	−6.8	NH-Arg120	F	H-bond (2.6 Å)
		Met522, Ser353	Furan ring	Pi–pi interaction
		Tyr355		
		Leu352, Val349, Ile523, Ala527, Leu531	Phenyl ring	Hydrophobic
			Isoxazole ring	
Compound **8**	−9.5	OH-Met522	NH	H-bond (2.4 Å)
			NH	H-bond (3.1 Å)
		Phe209	Phenyl group	Pi–pi interaction
		Phe381	Phenyl group	
		Tyr385	Pyrazole group	
		Tyr355	Pyrazole group	
		Trp387, Leu352	Benzofuran moiety	Hydrophobic
		Leu534, Leu535		

**Table 4:** Drug-likeness, Lipinski rule, and synthetic accessibility of compound **8**.

Drug-Likeness Parameters	Compound 8
Smile	[O+]=1c2c(CC=1c1nn(cc1C1=C(C#N)C(=O)N(N)C(N)=C1C#N)-c1ccccc1)cccc2
MW	434.438
Log P_o/w_(XLOGP3)	1.59
Log P_o/w_(MLOGP)	1.82
HBD	2
HBA	5
NRB	3
MR	121.36
Lipinski	Yes (0 violation)
SA	3.79
TPSA	137.75
Bioavailability score	0.55
PAINS filtrate	Yes (0 violation)

MW = molecular weight, log Po/w = lipophilicity, HBD = hydrogen bond donor, HBA = hydrogen bond acceptor, NRB = number of rotatable bonds, MR = molecular refractivity, SA = synthetic accessibility (from 1 (very easy) to 10 (very difficult)), TPSA = topological polar surface area.

**Table 5:** Pharmacokinetics characteristics of compound **8**.

Absorption		
Model Name	Predicted Value	Unit
Water solubility	−3.37	log mol/L
Human intestinal absorption (HIA)	89.13	%
Caco2 permeability	0.34	nm s^−1^
Skin permeability	−2.73	log Kp, cm h^−1^
Distribution		
BBB permeability	−0.54	log BB
CNS permeability	−2.37	log PS
Metabolism		
CYPC1A2 inhibition	No	
CYP2D6 inhibition	No	
CYP3A4 substrate	Yes	
Excretion		
Total clearance	1.08	log ml/min/kg
Renal OCT2 substrate	No	

HIA = human intestinal absorption (0–20 (poor), 20–70 (moderate), and 70–100 (well)), Caco2 = *in vitro* Caco2 cell permeability, BBB = blood–brain barrier permeability.

**Table 6:** Predicted toxicity using Data Warrior.

Test	Carcinogenicity (Mouse)
Mutagen	none
Tumorigenic	none
Irritant	none

## Data Availability

Not applicable.

## Supplementary Materials

The following supporting information can be downloaded at: https://www.mdpi.com/article/10.3390/molecules28196814/s1.

## Sample Availability

Samples of the compounds are available from the authors.
