# Supplementary material for: Development of a New Benzofuran–Pyrazole–Pyridine-Based Molecule for the Management of Osteoarthritis
Source: Molecules. 2023 Sep 27;28(19):6814. doi: 10.3390/molecules28196814 (PMC10574112; doi:10.3390/molecules28196814)
Supplement: Supplementary file 1 [file molecules-28-06814-s001.zip › molecules-2567188-supplementary.pdf]

Supporting information

Development of a New Benzofuran–Pyrazole–Pyridine-Based Molecule for the Management of Osteoarthritis

Somaia S. Abd El-Karim [1,*], Ahlam H. Mahmoud [1], Asmaa K. Al-Mokaddem [2], Noha E. Ibrahim [3], Hamad M. Alkahtani [4], Amer Alhaj Zen [5] and Manal M. Anwar [1,*]

[1] Department of Therapeutic Chemistry, Pharmaceutical and Drug Industries Research Division, National Research Centre (NRC), El Bohouth St., Dokki, Cairo 12622, Egypt; ahlamhosnynrc@gmail.com
[2] Department of Pathology, Faculty of Veterinary Medicine, Cairo University, Cairo 12211, Egypt; asmaa.khairy@cu.edu.eg
[3] Department of Microbial Biotechnology, Biotechnology Research Institute, National Research Centre (NRC), El Bohouth St., Dokki, Cairo 12622, Egypt; nohaelsayed855@gmail.com
[4] Department of Pharmaceutical Chemistry, College of Pharmacy, King Saud University, Riyadh 11451, Saudi Arabia; ahamad@ksu.edu.sa
[5] Chemistry & Forensics Department, Clifton Campus, Nottingham Trent University, Nottingham Ng11 8NS, UK; amer.alhajzen@ntu.ac.uk
* Correspondence: ssabdelkarim@gmail.com (S.S.A.E.-K.); mm.anwar@nrc.sci.eg (M.M.A.)

2. Experimental protocols

2.1. Chemistry

All melting points are uncorrected and were taken in open capillary tubes using Electrothermal apparatus 9100 (London, UK). Elemental microanalyses were carried out in the Microanalytical Unit, Central Services Laboratory, National Research Centre, Dokki, Cairo, Egypt, using Vario Elementar (Berlin, Germany) and were found to be within ±0.5%of the theoretical values. Infrared spectra were recorded on a Jasco FT/IR-6100 Fourier transform infrared spectrometer (Tokyo, Japan) using the KBr disc technique in the Central Services Laboratory, National Research Centre, Dokki, Cairo, Egypt. ^{1}HNMRspectra were determined by using a Jeol EX-270 NMR spectrometer (Tokyo, Japan) at the Central Services Laboratory, National Research Centre, Dokki, Cairo, Egypt, while ^{13}C NMR spectra were recorded using a Varian 300NMRspectrometer (Madison, WI, USA) at Marquette University, Milwaukee, WI, USA, and measured using TMS as an internal standard. The mass spectra were measured with a FinniganMATSSQ-7000 mass spectrometer (Olympia, WA, USA) at the Central Services Laboratory, National Research Centre, Dokki, Cairo, Egypt. Follow-up of the reactions and checking of the purity of the compounds were made by TLC on silica gel-precoated aluminium sheets (Type 60, F 254, Merck, Darmstadt, Germany) and the spots were detected by exposure to a UV lamp at 254 nm for a few seconds. The chemical names for the prepared compounds are

given according to the IUPAC system. 2-Acetylbenzofuran (**1**), 1-(benzofuran-2-yl)ethylidene-2-phenylhydrazone (**3**), 3-(benzofuran-2-yl)-1-phenyl-1*H*-pyrazol-4-carboxaldehyde (**4**), 2-((3-(benzofuran-2-yl)-1-phenyl-1*H*-pyrazol-4-yl)methylene)malononitrile (**6**) were prepared according to methods reported in the literature [34-38].

4.2.2.1. DPPH radical scavenging assay

Free radical scavenging ability of the extracts was tested by DPPH radical scavenging assay as described by Desmarchelier et al., 1997. The hydrogen atom donating ability of the compound was determined by the decolorization of methanol solution of 2,2-diphenyl-1-picrylhydrazyl (DPPH). DPPH produces violet/purple color in methanol solution and fades to shades of yellow color in the presence of antioxidants. A solution of 4 mg DPPH /10 ml methanol was prepared, and 125µL of this solution was mixed with 500µL of compound in methanol at different concentrations (10–1000 µg/mL). The volume was completed to 1ml by methanol. The reaction mixture was vortexed thoroughly and left in the dark at room temperature for 30 min. The scavenging potential was compared with a solvent control (0% radical scavenging) and the standard compound. Radical scavenging activity was calculated by the following formula:

% radical scavenging = [(A0 – A1) / A0] x 100,

where: A0 absorbance of control and A1 – absorbance of tested compound. The experiment was repeated three times at each concentration.

Supporting data

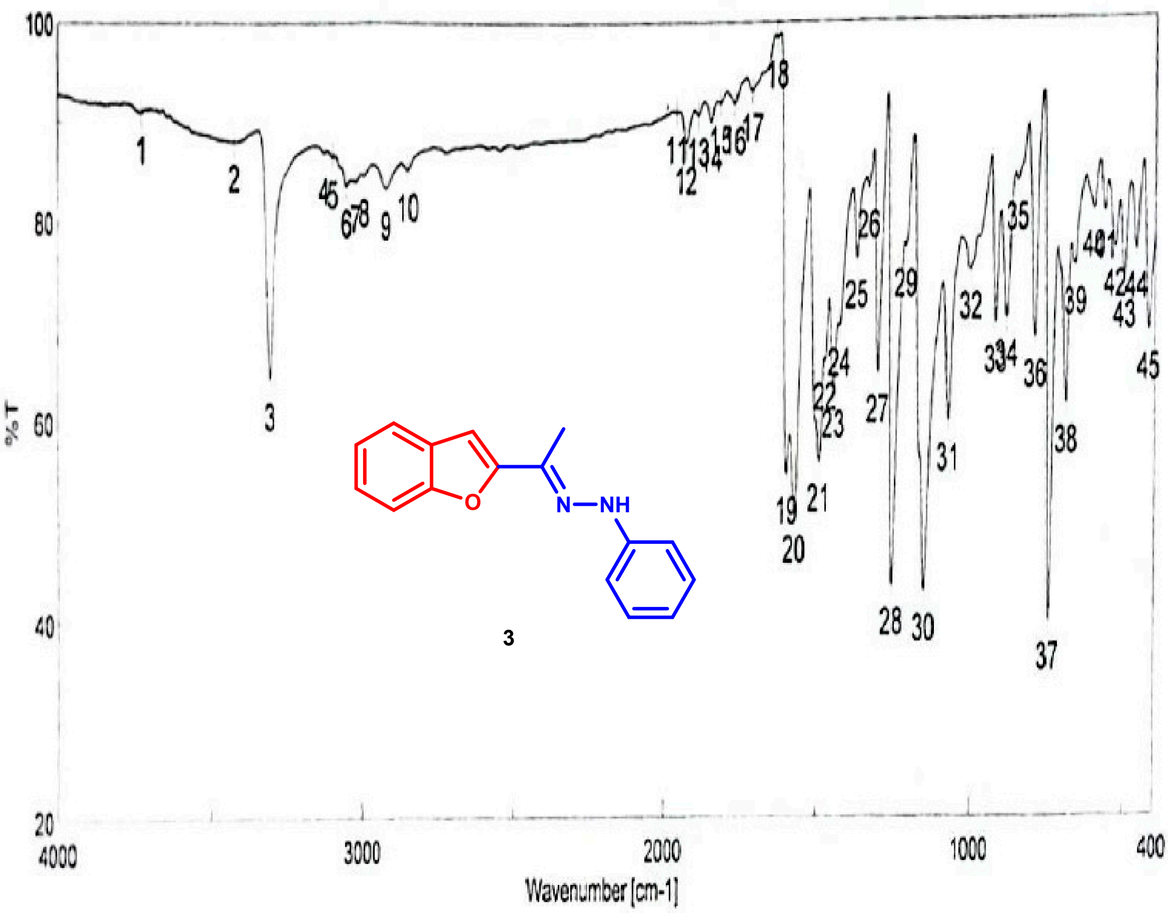

[Result of Peak Picking]

No.	Position	Intensity	No.	Position	Intensity	No.	Position	Intensity	No.	Position	Intensity
1	3727.73	90.9395	2	3423.99	87.9864	3	3309.25	64.605	4	3130.87	86.9382
5	3102.9	86.5911	6	3056.62	83.7518	7	3027.69	84.2165	8	2999.73	84.8525
9	2927.41	83.5899	10	2855.1	85.4145	11	1967.04	91.0602	12	1936.18	87.9727
13	1896.65	90.7714	14	1854.22	90.1675	15	1824.33	91.9834	16	1777.08	91.8558
17	1720.19	93.1327	18	1640.16	98.4242	19	1599.66	54.7531	20	1573.63	50.8773
21	1494.56	55.9776	22	1475.28	66.0918	23	1448.28	63.3519	24	1428.99	69.4809
25	1374.03	76.2033	26	1335.46	83.1857	27	1303.64	64.7671	28	1254.47	43.5169
29	1214.93	77.2378	30	1149.37	42.837	31	1074.16	59.8481	32	1002.8	74.8813
33	921.807	69.4982	34	887.095	70.0115	35	852.382	83.7688	36	793.564	67.9994
37	746.317	39.7391	38	692.32	61.4014	39	663.393	75.2141	40	601.682	80.7812
41	566.005	80.5868	42	533.221	76.8489	43	502.366	73.7401	44	462.832	76.5998
45	421.37	68.5523									

Figure S1: IR spectrum of compound 3

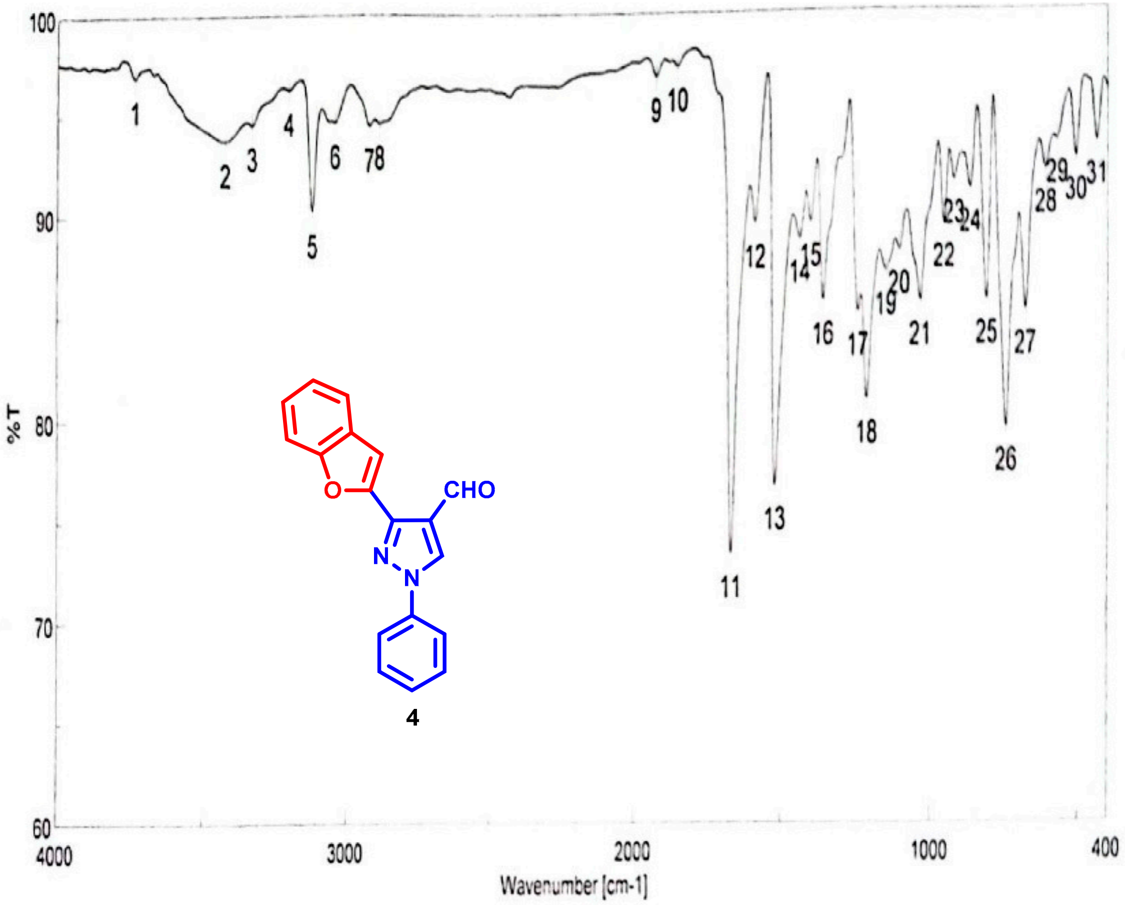

[Result of Peak Picking]

No.	Position	Intensity	No.	Position	Intensity	No.	Position	Intensity	No.	Position	Intensity
1	3733.51	96.9365	2	3420.14	93.7238	3	3328.53	94.5223	4	3201.26	96.2585
5	3121.22	90.4457	6	3042.16	94.7762	7	2923.56	94.6404	8	2888.84	94.7819
9	1937.15	96.9135	10	1864.83	97.3768	11	1669.09	73.3757	12	1595.81	89.599
13	1522.52	76.7203	14	1444.42	88.8196	15	1408.75	89.6049	16	1364.39	85.7435
17	1247.72	85.1712	18	1216.86	80.8629	19	1154.19	87.1358	20	1108.87	88.1335
21	1036.55	85.621	22	956.52	89.4191	23	922.771	91.5529	24	866.846	91.135
25	811.885	85.647	26	745.352	79.3819	27	679.785	85.1147	28	609.396	92.0087
29	570.626	93.4824	30	507.187	92.5973	31	436.798	93.3357			

Figure S2: IR spectrum of compound 4

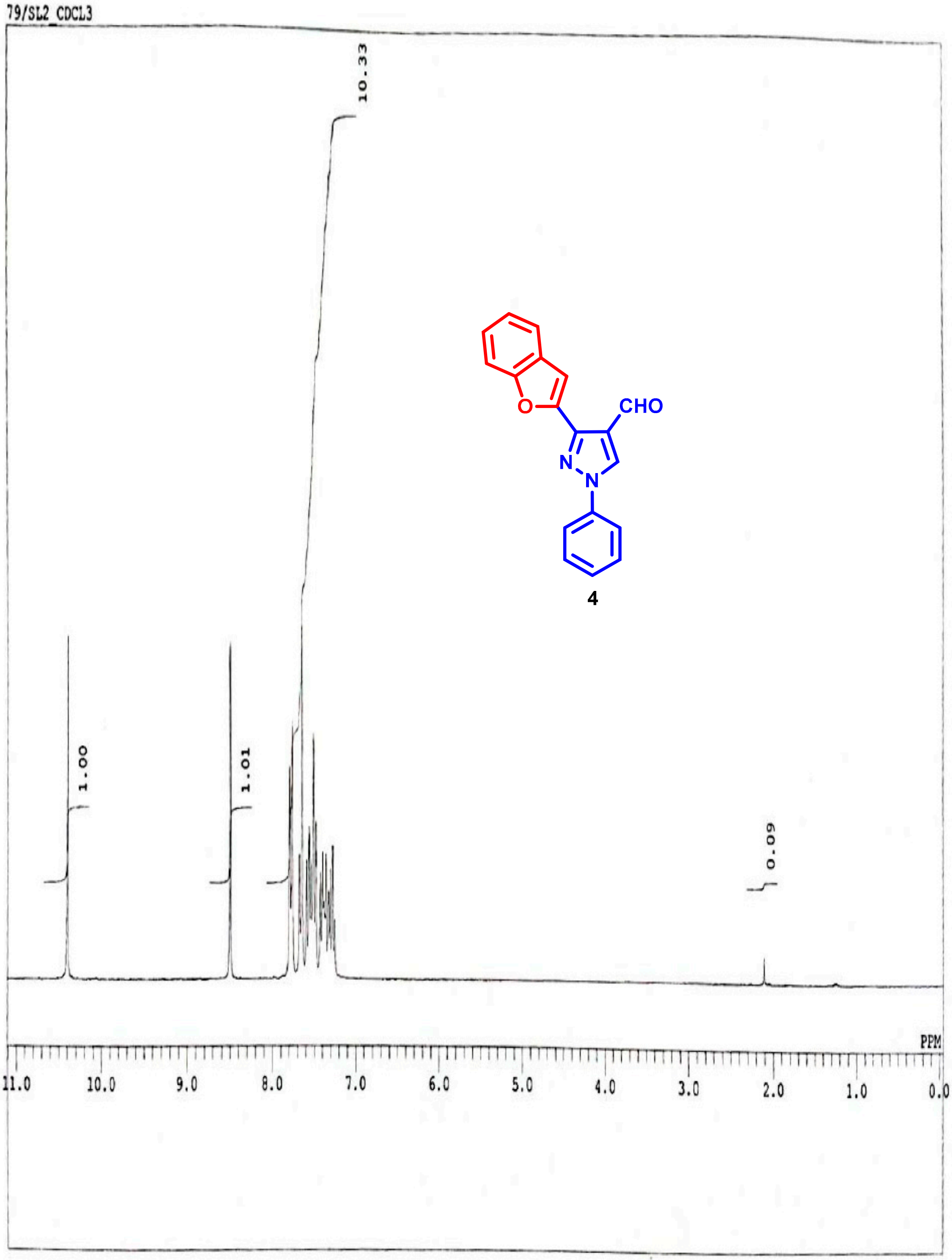

Figure S3: ^{1}H-NMR spectrum of compound 4

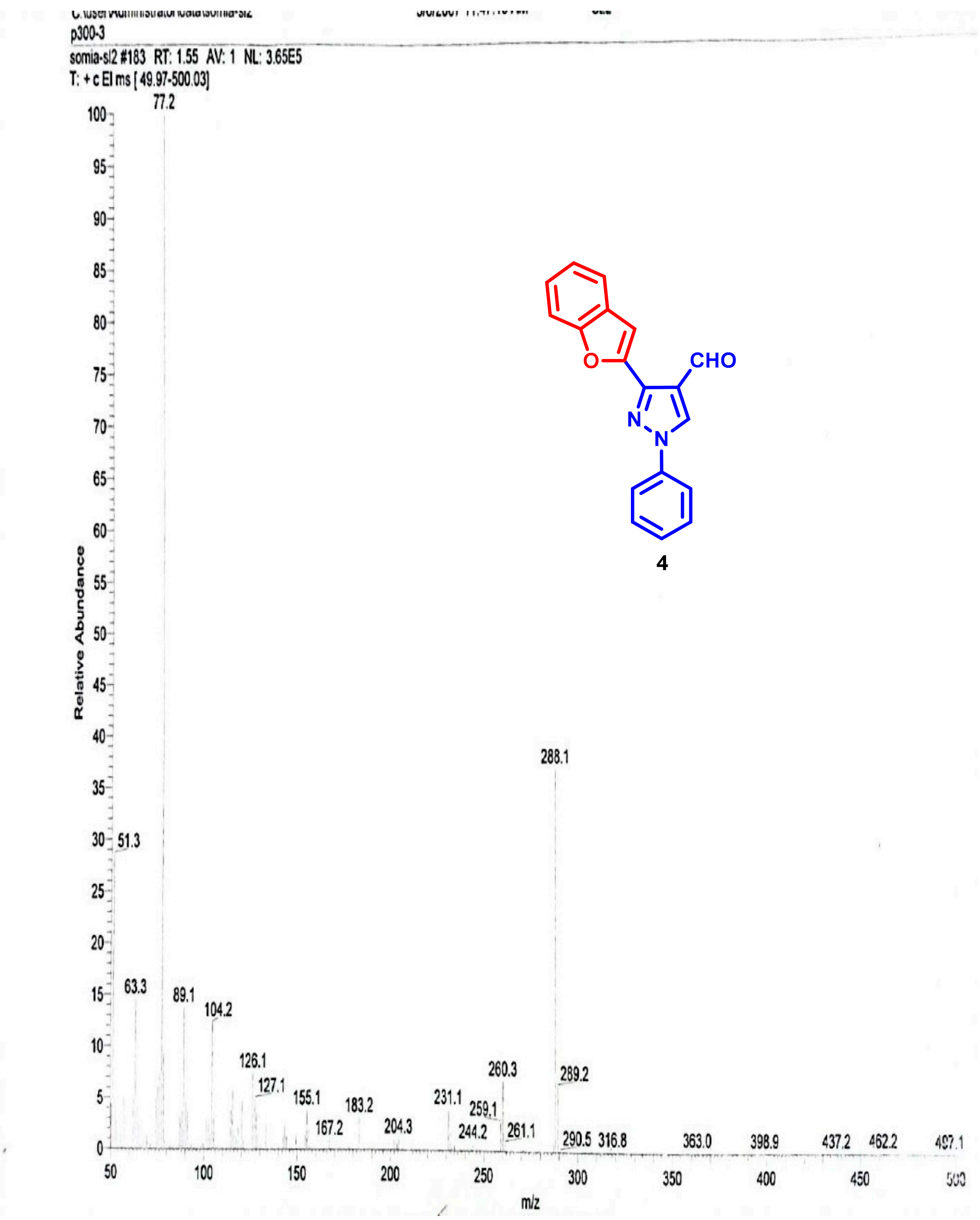

Figure S4: Mass spectrum of compound 4

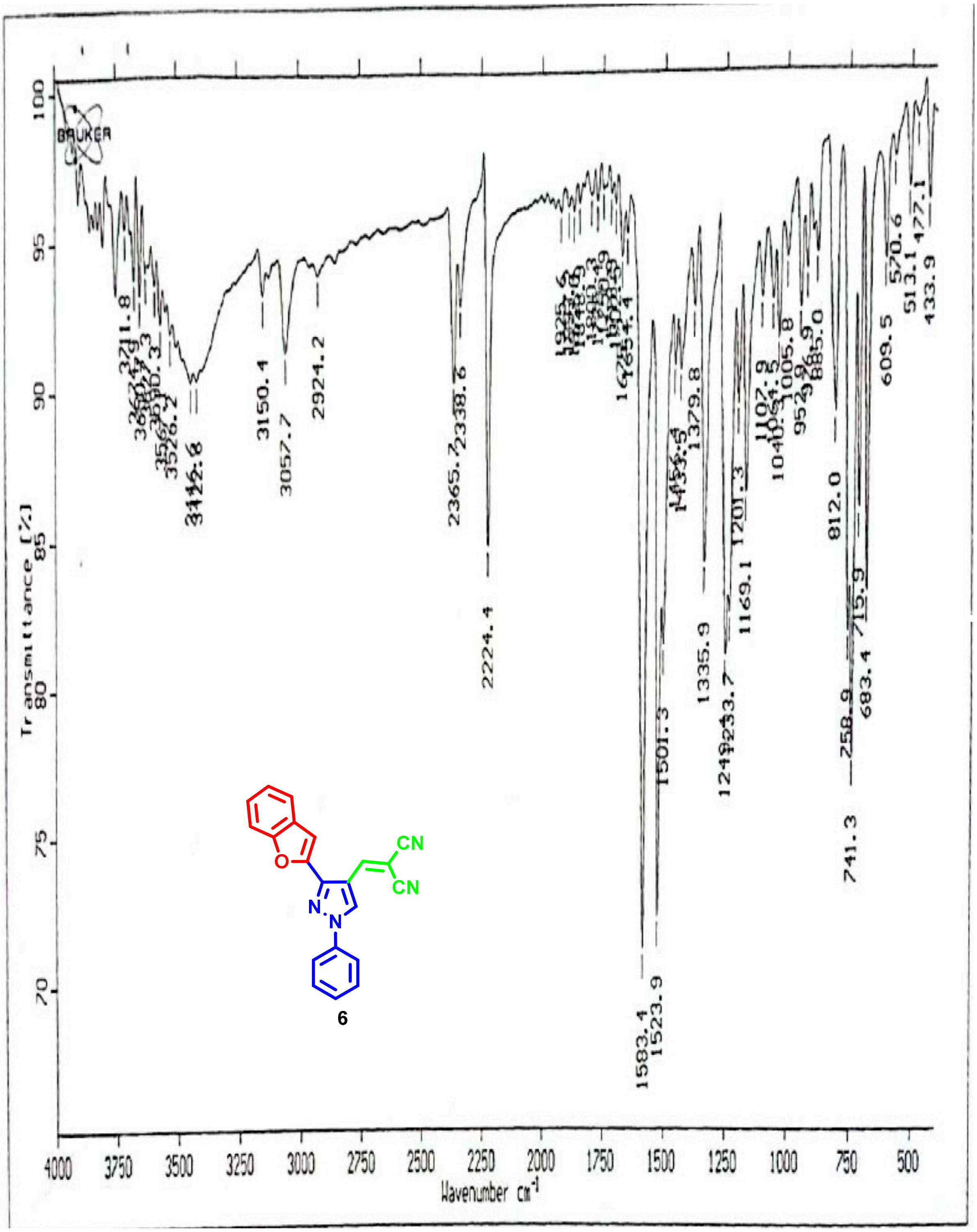

Figure S5: IR spectrum of compound 6

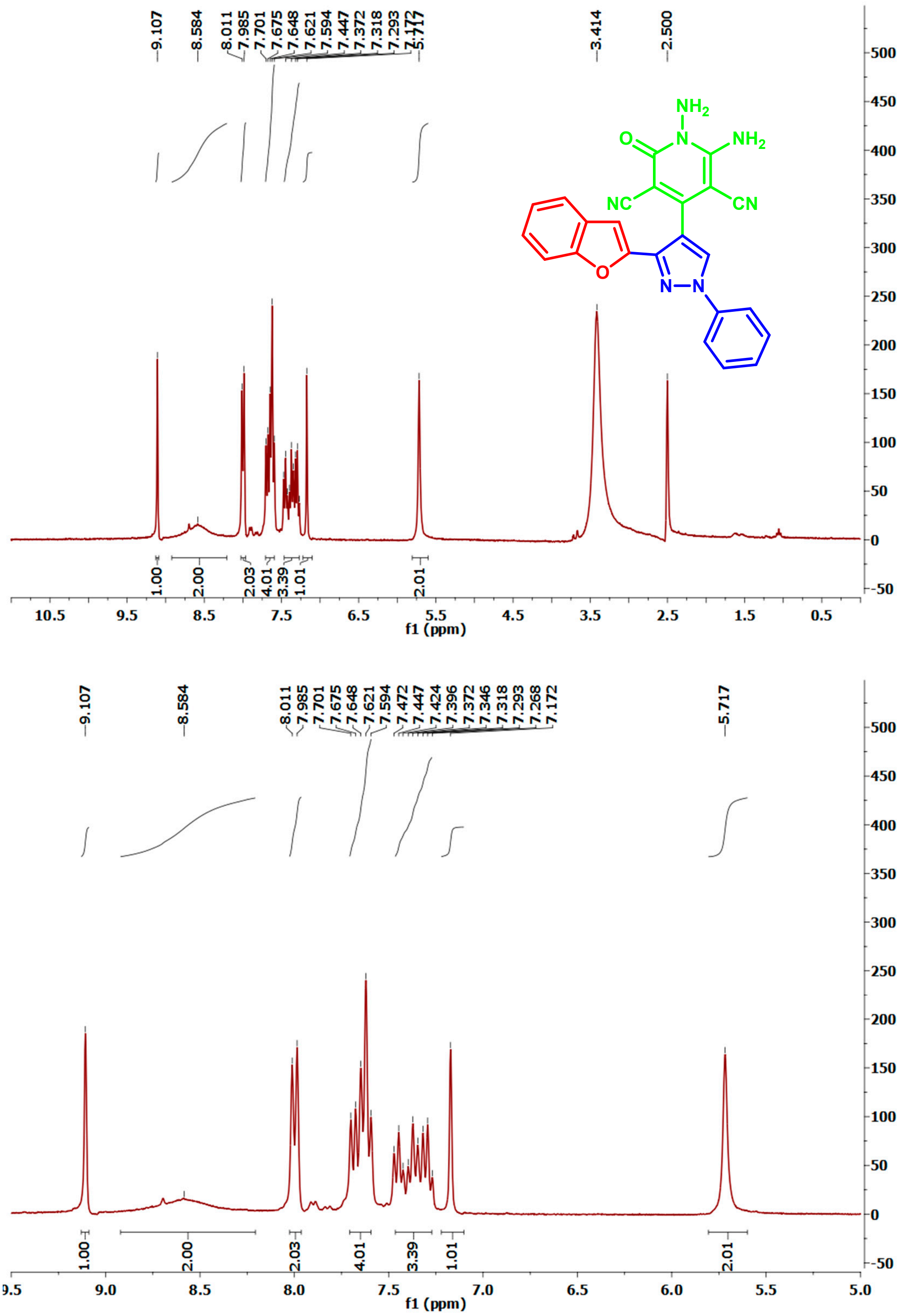

Figure S6: ^{1}H-NMR spectrum of compound 8

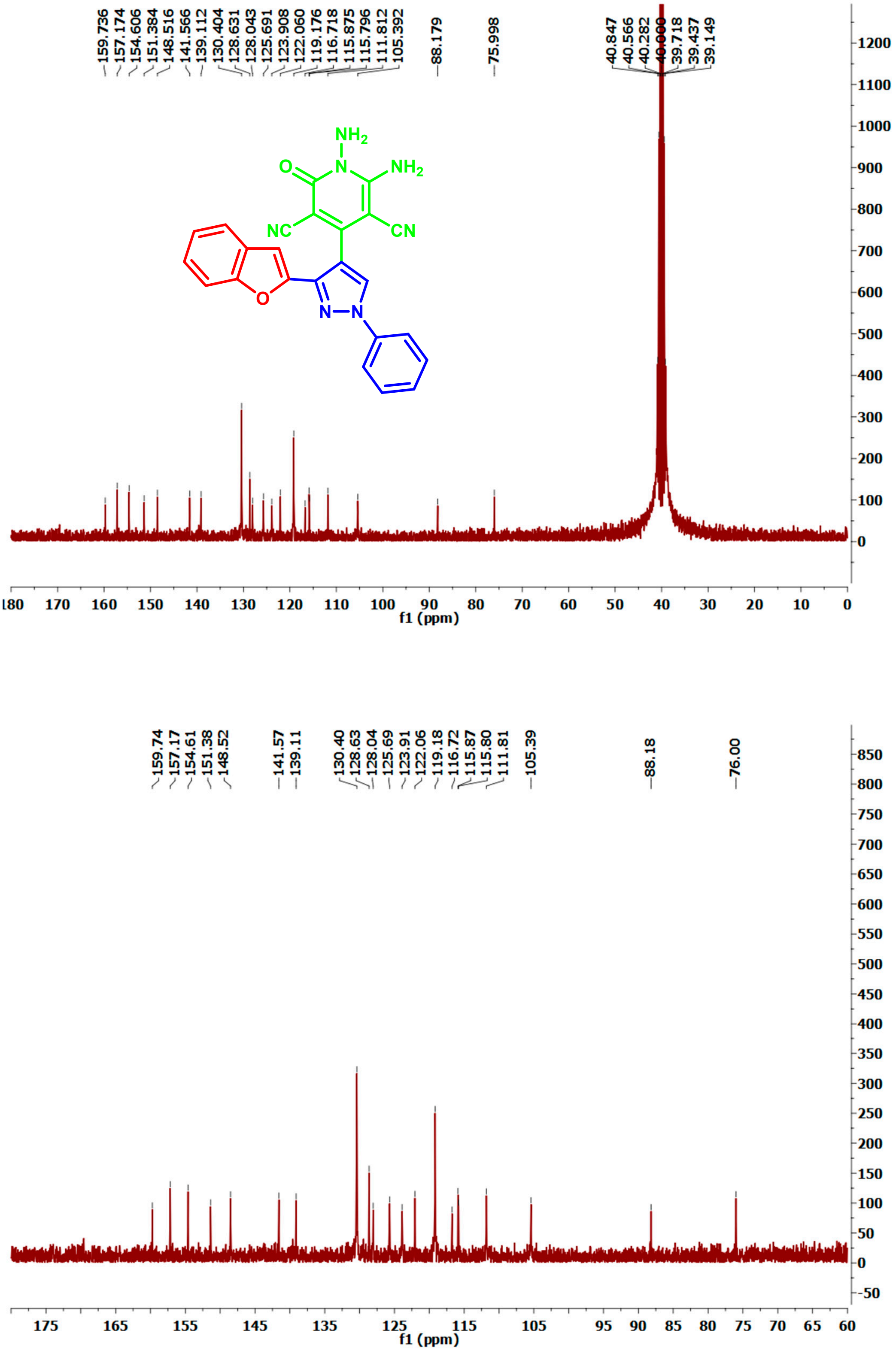

Figure S7: ^{13}C-NMR spectrum of compound 8
